# Editorial: Advancing HIV treatment and prevention for cisgender women: approaches to optimize health outcomes

**DOI:** 10.3389/fmed.2026.1797624

**Published:** 2026-02-18

**Authors:** Maria L. Alcaide, Renee Heffron, Seble G. Kassaye, Kathleen M. Powis, Anandi N. Sheth

**Affiliations:** 1 University of Miami MIller School of Medicine, Miami, FL, United States; 2 University of Alabama, Birmingham, AL, United States; 3 Georgetown University Medical Center, Washington, DC, United States; 4 Harvard Medical School, Boston, MA, United States; 5 Emory University School of Medicine, Atlanta, GA, United States

**Keywords:** HIV, HIV prevention, HIV treatment, reproductive health, women

The landscape of HIV treatment and prevention options is rapidly evolving, yet the global burden of HIV remains disproportionately high for cisgender women, who account for half of all infections worldwide. This editorial introduces the Research Topic “*Advancing HIV Treatment and Prevention for Women*,” a collection of multidisciplinary studies and perspectives illuminating the unique challenges, innovations, and opportunities for improving health outcomes among women living with (WWH) and women needing HIV prevention globally. The articles published in this Research Topic highlight the hurdles faced by women, not only for receiving optimal HIV treatment and prevention, but also for critical reproductive and sexual health services. They underscore the urgent need for tailored, evidence-based strategies and interventions that address the complex interplay of factors across communities, contexts, and life stages that shape the experiences of women affected by HIV seeking HIV and reproductive and sexual health services.

## Understanding the unique challenges facing women for HIV treatment and prevention

Women's vulnerability to HIV is shaped by a confluence of physiological, behavioral, and societal factors that also affect the success of HIV treatment and prevention. The articles in this Research Topic highlight the necessity of considering these factors, which are often intersecting, when addressing treatment and prevention for women.

Trauma and violence are pervasive among women living with HIV, with profound implications for mental health and engagement in care. Nazaire et al. evaluated the performance of trauma-informed care for refugee women living in the U.S. and advocate for the routine integration of trauma-informed, culturally responsive approaches within HIV care settings. The high prevalence of violence perpetrated by intimate partners and others, hate crimes, and adverse childhood experiences among WWH underscores the necessity of screening for violence and providing tailored support services. They propose that by centering women's voices and preferences, healthcare systems can foster safer, more empowering environments that promote holistic wellbeing. Garbarino et al. evaluated violence experienced by women in Atlanta, USA using structured surveys and in-depth interviews. Results revealed that experiences of violence are common among WWH and go beyond intimate partner violence to include non-partner violence, hate crimes, and adverse childhood experiences. These experiences were strongly associated with mental health conditions and negatively affected HIV care outcomes, again underscoring the need for trauma-informed approaches in HIV care. In this cohort, participants expressed a range of opinions for how to tailor violence screening and support services, noting positive and uncomfortable past experiences that informed their suggestions.

Psychosocial and behavioral factors have been linked to comorbidities in the general population and may play a larger role among WWH as they manage HIV as a chronic infection. Findings from Wise et al. revealed that adverse socioeconomic and psychosocial factors such as stigma, inadequate social support, and area-level deprivation contribute to changes in blood pressure and hypertension. In their study, both WWH and without HIV had high rates of uncontrolled blood pressure, but the factors influencing these outcomes varied by HIV status, emphasizing the need for interventions that address both individual and societal stressors.

Currently, HIV treatment and prevention are being transformed by the introduction of long-acting antiretroviral agents. A qualitative study by Hassan et al. in Kenya explored perceived benefits and barriers to long-acting antiretroviral therapy among adolescents and young people living with HIV in western Kenya identifying strong enthusiasm for the promise of long-acting agents to improve adherence, increase convenience, and reduce stigma. However, their results also highlight potential challenges within the health system that may impede the use of injectables, such as more frequent in-person visits for injections, changes to existing workflows and supply chain mechanisms, and the potential for injection-related adverse events that require further community sensitization and education to improve acceptability and ensure effective implementation.

Globally, PrEP use among women has lagged behind other populations at risk for HIV such as men who have sex with men. In another qualitative study in Kenya among young women who had declined, delayed, discontinued, or restarted PrEP, Ogello et al. found that. PrEP decisions were shaped by shifting HIV risk dynamics, one's sense of autonomy, partner influence, pill-related challenges, and low self-efficacy. Similar to Hassan et al., many participants expressed a preference for long-acting injectable PrEP due to its ease of use, privacy, and perceived support for adherence.

### Reproductive and sexual health and HIV

Reproductive health is intricately linked to HIV prevention and care for women. The analysis of pregnancy and contraceptive use among participants in the HVTN 705 vaccine trial in southern Africa by Mda et al. provides valuable insights for future trial designs and contraception. Findings reveal low contraception use, with about only 60% of participants reported contraceptive use, and a pregnancy rate of 2.95 per 100 person-years. Despite requirements to avoid pregnancy during the vaccination period, pregnancies occurred, with younger age and oral contraceptive use associated with higher incidence, with the lowest rate of pregnancies among those using contraceptive implants. These findings highlight the importance of integrating effective contraceptive counseling and access into HIV vaccine trials, particularly when younger women are a priority for participation.

The study by Latham et al. utilized electronic records in a single center in the US to evaluate retention in HIV care after pregnancy. Findings highlighted the challenges that women face with HIV care during the postpartum period when life with a newborn is particularly demanding. Among 182 pregnancies between 2019 and 2023, only one-third of moms were able to attend two postpartum visits more than 90 days apart and about one-third of participants were not virally suppressed 12 months after delivery. Not surprisingly, engagement in prenatal and postpartum obstetric care was associated with better HIV outcomes, amplifying the need for innovative, supportive interventions that facilitate the transition from obstetric to HIV care in the postpartum period to ensure sustained viral suppression and improved maternal and infant health.

Sexually transmitted infections (STIs) remain a significant concern for women of reproductive age, with implications for both HIV acquisition and reproductive health. The study by Nogueira et al. describes a cross-sectional analysis of genital and extragenital STIs among women with and without HIV in the Southern U.S who were enrolled in the STAR cohort. Their data reveals a high prevalence of STIs, particularly for trichomoniasis, chlamydia, and gonorrhea. Importantly, HIV status was not associated with STI prevalence, whereas lack of healthcare access, lower educational attainment, and lower income were associated with higher STI prevalence. These findings highlight the importance of comprehensive, women focused screening and prevention strategies that address socio-structural issues and health care access, especially in high-prevalence regions of the U.S.

### Innovative models and interventions

Two articles in this Research Topic present innovative models and interventions aimed at improving outcomes for women. In a cluster-randomized study in Lesotho, Greenberg et al. evaluated the impact of a facility-based intervention package on prevention of vertical HIV transmission and maternal-child health outcomes, enrolling 614 pregnant women with HIV and 390 without HIV across 12 facilities. The intervention, which included multidisciplinary care teams, patient-centered training, and home support, significantly improved adherence to antiretrovirals among WWH and increased HIV re-testing among those without HIV who need prevention. Findings suggest that integrating coordinated, patient-centered services that include home support within existing health systems is a scalable strategy to enhance maternal and child health outcomes. Finally, Pratt et al. described how the “Camellia Cohort” in Alabama proposes to leverage mHealth technology and community engagement to support sexual health among women, offering a promising model for remote delivery of tailored prevention and care in high-priority regions. Participants in the study completed home-based HIV/STI testing and online surveys every 6 months for up to 42 months. With data collected through testing, surveys, medical record review, and dried blood spots, the study aims to measure HIV and STI incidence, PrEP use, and key predictors of prevention behaviors, to provide insights into the feasibility and acceptability of the intervention.

### The path forward

The articles in this Research Topic collectively advance our understanding of the multifaceted and interlinked challenges and opportunities to optimize HIV treatment and prevention for women. This body of work provides evidence for the benefits of a holistic, intersectional approach that integrates biomedical, behavioral, and structural interventions and centers women's voices and experiences across reproductive stages to address the broader determinants of health while supporting HIV treatment and prevention. As the field moves forward, continued collaboration among researchers, clinicians, policymakers, and communities remains essential to drive progress and achieve equitable, optimal health outcomes for all women affected by HIV.

